# Ultrastructural Study on Tissue Alterations Caused by Trypanosomatids in Experimental Murine Infections

**DOI:** 10.3389/fpubh.2014.00075

**Published:** 2014-07-08

**Authors:** Héctor J. Finol, Antonio Roschman-González

**Affiliations:** ^1^Center for Electron Microscopy, Faculty of Sciences, Central University of Venezuela, Caracas, Venezuela

**Keywords:** pathology, ultrastructure, murine tissues, experimental infections, trypanosomatids

## Abstract

The ultrastructural study in different tissues of mice experimentally infected with isolates of *Trypanosoma evansi, Trypanosoma cruzi*, and *Leishmania mexicana* reveals changes in cardiac myocytes, skeletal muscle fibers, and hepatic, adrenal, kidney, and spleen cells. Some of these changes were cytoarchitectural and others consisted of necrosis. Alterations in the microvasculature were also found. The mononuclear cell infiltrate included neutrophils, eosinophils, and macrophages. This work shows that diverse mice tissues are important target for trypanosomatids.

## Introduction

At the present time, there is a vast literature concerning the effects of protozoan parasites in the ultrastructure of mammalian tissues. These works include horse and mouse skeletal muscles infected by *Trypanosoma evansi* (1, 2) and *Toxoplasma gondii* (3, 4), human skeletal and mouse cardiac muscles by *Trypanosoma cruzi* (5, 6), avian skeletal muscle by *Plasmodium cathemerium* (7), and human skeletal muscle by *Plasmodium falciparum* (8). Alterations in the mouse adrenal gland and liver were provoked by *Trypanosoma evansi* (9, 10) and *Plasmodium berghei* (11, 12). In this context, it would be interesting to know if structural changes observed are similar in all studied species. Furthermore, with this investigation we intend to perform a systematic work on the ultrastructure of alterations in experimental murine infections by some trypanosomatids. This could help to understand better the biology of trypanosomatids in vertebrate host.

## Materials and Methods

### Experimental infections

For experimental infections, Balb/c mice were used. They were divided into 3 groups of 10 mice each one for the three used species (*Trypanosoma cruzi, Trypanosoma evansi*, and *Leishmania mexicana*), and one additional 10-mice group was used as a control. Animals were infected by intraperitoneal (i.p.) route with inocula consisting of 10 parasites/g of animal body weight and uninfected mice were kept as controls. Three mice from each group were randomly selected after prepatent period and killed under anesthesia during peaks of infection. Then tissue samples were removed and processed for transmission electron microscopy. The experimental procedures were approved by the ethical committee of the Sciences Faculty at the Central University of Venezuela, and the work was conducted in agreement with the regulatory standards.

### Transmission electron microscopic study

Tissue samples were fixed with Karnovsky’s solution, in phosphate buffer at pH 7.4 and 320 mOsm, post-fixed in 1% O_s_O_4_, and embedded in epon resin. Sections were cut with a diamond knife in a Porter-Blum MT-2B ultramicrotome and stained with uranyl acetate and lead citrate. Ultrathin sections were observed in a Jeol JEM – 1011 transmission electron microscope, at an accelerating voltage of 80 kV.

## Results

**Figure 1:**
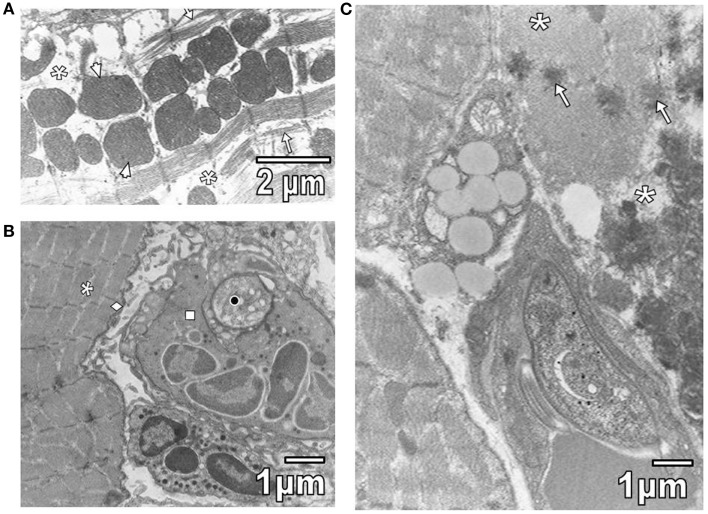
**(A)** This section shows widened intermyofibrillar spaces (asterisks) of cardiac myocytes in *T. evansi* extensively occupied by mitochondria (arrowheads) and disorganized sarcomeres (star). **(B)** Section of skeletal muscle from a mouse parasitized with *L*. *mexicana* is shown. Intermyofibrillar (asterisk) and subsarcolemmal (rhombus) spaces are slightly widened. Observe a parasite (black circle) inside of neutrophil (square). **(C)** Section of skeletal muscle from a mouse parasitized by *T. evansi* is shown. Note areas of segmental necrosis (asterisks) showing mitochondrial debris with paracrystalline inclusions (arrows).

**Figure 2:**
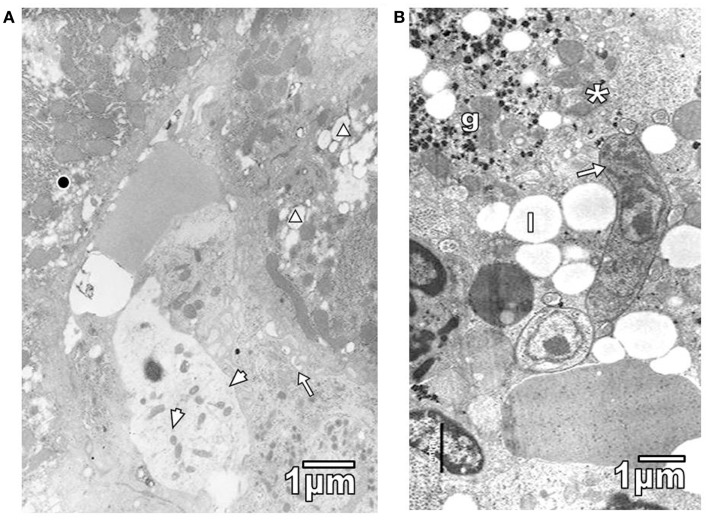
**(A)** Section of hepatic parenchymatous cells from a mouse parasitized with *T*. *cruzi* is shown. Observe lipid droplets (triangle), glycogen depleted areas (black circle), some microvilli in Disse space (arrow), and widened sinusoid endothelial cells with few pinocytic vesicles (arrowheads). **(B)** Sinusoid lumen showing *T. evansi* parasite (arrow) and hepatocyte debris, including glycogen particles (g), mitochondria (asterisk), and lipid droplets (l).

**Figure 3:**
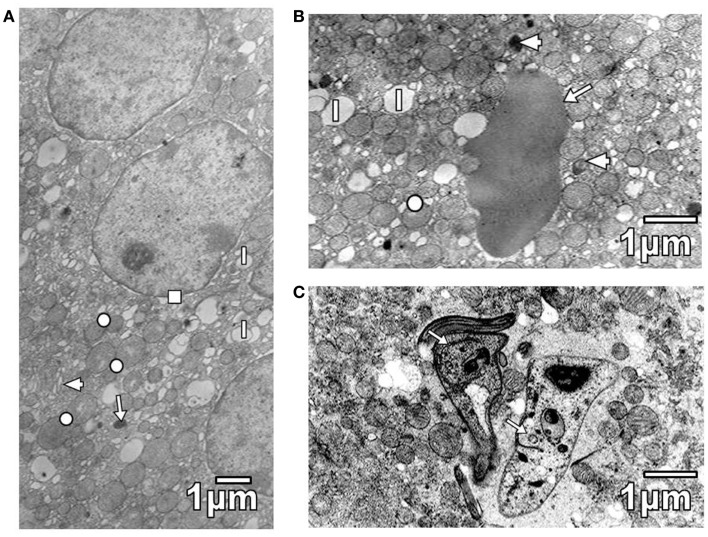
**(A)** Section of cortical cells shows swollen elements of SER (arrowhead), some mitochondria with decreased cristae (circle), lipid droplets (l), lysosomes (arrow), and a widened nuclear envelop (square). **(B)** Section of a cortical cell from *T. cruzi* parasitized mouse, showing mitochondria with variable number of cristae (circle), lipid droplets (l), and lysosomes (arrowheads). Note the presence of an erythrocyte (arrow). **(C)** Section of a cortical cell from a mouse parasitized with *T. evansi* is shown. Observe the presence of parasites (arrows).

Pathological reactions in experimentally infected mice with diverse trypanosomatids showed some common characteristics in studied tissues of different replicates for each experimental group. Cardiac myocytes in *T. evansi* (Figure 1A) and skeletal muscle fibers in *T. evansi* and *L. mexicana* (Figure 1B) parasitized animals exhibited atrophy. In cardiac myocyte sections, myofibrillar disorganization and myofilament loss were seen (Figure 1A). Skeletal muscle fibers from mice infected with *T. evansi* showed segmental necrosis. In these areas (Figure 1C), mitochondrial paracrystalline debris was located next to contractile masses. As it is showed in Figure 2A, in *T. cruzi* similarly to the case of infection with *T. evansi*, liver hepatocytes showed an increment of lipid droplets, depletion in glycogen content, and decrease of microvilli in Disse’s space. Sinusoid endothelial cells were widened with scarce pinocytotic vesicles. Hepatocyte debris was observed in some sinusoids, suggesting parenchymatous cell necrosis (Figure 2B). In mice infected by different trypanosomatids, adrenal cortical cells alterations were represented by lack of cytoarchitectural relations between mitochondria and smooth endoplasmic reticulum (SER), swelling of SER elements, decrease of mitochondrial cristae, widened nuclear envelop, change of electron density in cell cytoplasm, and presence of lysosomes (Figures 3A,B). Intracellular erythrocytes were observed in the infection with *T. cruzi* (Figure 3B), while *T. evansi* parasites were seen in cortical cells of parasitized mice (Figure 3C). Additionally, capillary fenestrae were widened.

**Figure 4:**
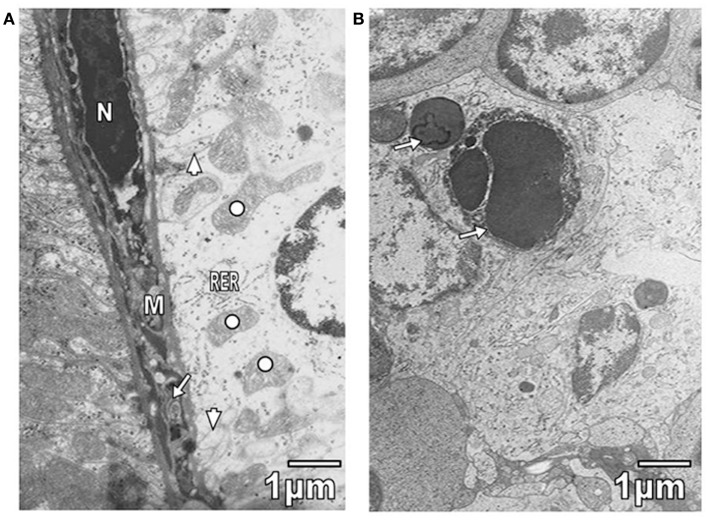
**(A)** Section from proximal convolute tubule of a mouse parasitized by *T*. *evansi* is shown. Observe portions of interdigitations (arrowheads), swelling of RER cisternae, and mitochondrial cristae (open circle). In the capillary, swollen RER (arrow), mitochondria (M), and hyperchromatic nucleus (N) are seen. **(B)** Section of spleen from parasitized mouse with *T. evansi* is shown. Note the presence of apoptotic bodies (arrows).

**Figure 5:**
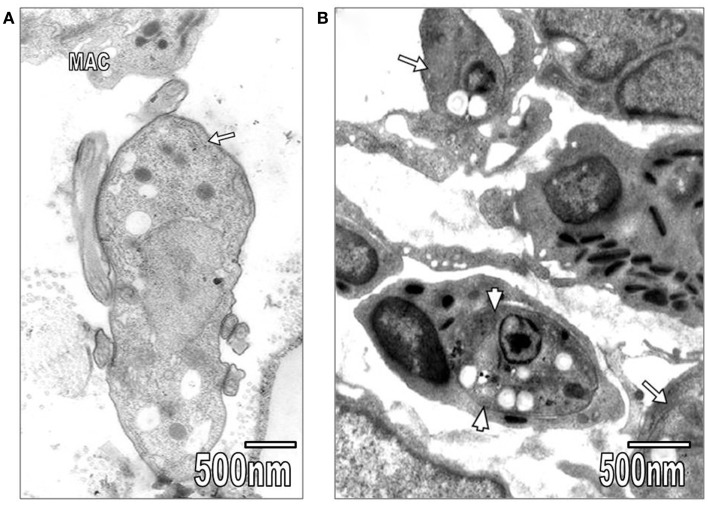
**(A)** In this section, *T*. *evansi* (arrow) and a macrophage (MAC) are seen. **(B)** Section showing *L. mexicana* organisms inside of an eosinophil (arrowheads) and a macrophage (arrows).

Kidney convoluted tubules were observed with thickened basement membrane, disorganization of interdigitations, and significant decrease of their number; in some areas was noted swelling of rough endoplasmic reticulum (RER) and mitochondrial cristae (Figure 4A). As it is seen in Figure 4A, the capillary endothelial cell cytoplasm also presented swollen RER and mitochondria. Spleen ultrastructure was studied in *T. evansi-*infected mice. Tissular disorganization, fibrosis, and apoptotic bodies (Figure 4B), as well as necrosis were observed. The inflammatory infiltrate consisted of mononuclear cells, such as neutrophils, eosinophils, and macrophages (Figures 1B and 5A,B). Trypanosomatids were found in extracellular spaces and inside of mononuclear cells (Figures 1B and 5A,B).

## Discussion

Infection with several trypanosomatids leads to a rapidly lethal disease in different strains of mice. According to various authors [for review Ref. (13)], living and dead trypanosomes produce a number of biologically active substances, which are involved in the etiology of lesion. As it has been shown in mice infected with *T. cruzi*, the acute infection is characterized by a severe immune depression (14). Immunosuppresion also occurs in Leishmaniasis (15). The mechanism of action of released molecules by *T. cruzi* and *Leishmania* sp. could suggest a role as regulatory activating and inhibiting factors of host immune cells (14, 16, 17). The role of IFN-γ during *T. cruzi* infection was demonstrated when IFN-α and IFN-γ receptor KO mice showed higher rates of parasitemia and mortality (18). Infected IFN-γ KO mice showed increase in cellular infiltrates in heart and skeletal muscles and reduced survival (18–20).

A number of reports have documented the role of NO in host defense against pathogens. In the case of *T. cruzi*, experimental infection induces NO production and suggests that IFN-γ and TNF-α are involved in the phenomenon (21, 22). Recently, it was reported that in *L. amazonensis* infected mice, pravastatin increased the phagocytosis mediated by complement and immunoglobulin receptors, and induced a rise of nitric oxide production by macrophages, allowing these cells to kill ingested leishmania organisms, with reduction of the overproduction of tumor necrosis factor (23). Other experiments have shown that IFN-γ and TNF-α-mediated activation of macrophages leads to increased production of NO, which in turn suppresses T cell activation. NO and oxygen radicals release from locally activated macrophages and stimulated endothelial or tissue cells, have been implicated as the final mediators in cytokine-induced pathology in malaria (24, 25).

Our investigation in murine experimental infection with trypanosomatids is in line with a degree of striated muscle alterations, which varied from slight to severe, during the pathogenesis and development of disease. The ultrastructural pathology data are similar to previous results concerning cardiac myocytes in hamsters and mice experimentally infected with *T. cruzi* (6, 26), “derrengadera” by *T. evansi* in wild horse skeletal muscle fibers (1), and in mice parasitized with *T. gondii* (3). In advanced Chagas’ disease patients, capillary damage also has been reported in skeletal muscle (5).

These results in relation to changes in liver hepatocytes of mice infected by *T. cruzi* and *T. evansi* were in some aspects similar to those described in liver of mice parasitized with *P. berghei* (27), including an increment of lipid droplets and depletion of glycogen particles, simultaneously with a decrease of microvilli in the Disse’s space. Also, necrotic hepatocytes and a thickening of endothelial cell cytoplasm were found in both cases. In adrenal cortex of mice infected with *P. berghei* (11), erythrocytes were observed inside cortical cell cytoplasm, as we also observed in the infection with *T. cruzi* and the report by Rodríguez-Acosta et al. (28) in cortex of adrenal gland in mice injected with a lethal dose fifty (LD_50_) of bee venom. In the investigation of Pulido-Méndez et al. (11), parasites were not seen inside of cortical cells. On the contrary, in *T. evansi* infected cortical cells contained trypanosomes as it was described by Rossi et al. (9), and in the present work.

The ultrastructural pathological changes as those described in hepatocytes and adrenal cortical cells also were found in kidney of mice in *Plasmodium berghei* infection (12), including swelling of some organelles and disorganization of interdigitations and decrease of their number in some areas. Interestingly, loss of interdigitations and tubular vacuolization were also described in convoluted proximal tubules of mice intraperitoneally injected with a lethal dose fifty LD_50_ of *Apis mellifera* (29) in association with swelling of endothelial cell mitochondria and RER as in the present work.

Besides the splenic changes caused by action of parasite could be related to a possible capability for particular proteolytic secretions (9), due to a *T. evansi* induced hepatic alteration since a liver deterioration can rise the portal pressure. Indeed, advanced hepatomegaly increases the portal flux causing the blood to flow through collateral systems via portal and cava veins (30). The portal flux increments are determined by vasodilatation of the splanchnic tissue (stomach, intestine, pancreas, and spleen) admitting consequently an augmentation in the blood flux arriving to the organs (31). The first ultrastructural indication of damage is through a considerable amount of splenic debris. According to Jain (32), the presence of such remains is derived from erythrophagocytosis and cell debris phagocytosis occurring in the infected spleen.

The mononuclear cell infiltrate consisted of neutrophils, eosinophils, and macrophages. Macrophages and eosinophils were reported by Tonino et al. (3) in mice infected by *T. gondii*. Similarly, macrophages were reported by Quiñones Mateu et al. (1) in horses parasitized by *T. evansi* and in mice infected by *P. berghei* (12). In our investigation, we did not observe mastocytes as described in the infection with *T. gondii* (3) and lymphocytes as reported in mice infected by *P. berghei* (12). Our ultrastructural study demonstrates that several tissues of mice are certainly targets for trypanosomatids. Moreover, the murine model is very useful for pathological studies in trypanosomiasis using transmission electron microscopy.

## Conflict of Interest Statement

The authors declare that the research was conducted in the absence of any commercial or financial relationships that could be construed as a potential conflict of interest.
